# P077: Observations on incidence of quinolone resistance genes and their association with SHV genotypes and bacterial sequence type in a Klebsiella pneumoniae outbreak

**DOI:** 10.1186/2047-2994-2-S1-P77

**Published:** 2013-06-20

**Authors:** HH Balkhy, S Aljohani, A Almasood, T Uzzaman

**Affiliations:** 1 Infection Prevention and Control, King Abdulaziz Medical City, NGHA, Riyadh, Saudi Arabia; 2 Microbiology, King Abdulaziz Medical City, NGHA, Riyadh, Saudi Arabia; 3 King Abdullah International Medical Research Center, King Abdulaziz Medical City, NGHA, Riyadh, Saudi Arabia

## Introduction

Plasmid-borne genes conferring quinolone resistance have been increasingly recognized in Klebsiella pneumoniae (Kp) infections.

## Objectives

To explore multidrug resistance, we analyzed the presence of quinolone resistance genes in combination of other ESBL genes in isolates resulting from a hospital outbreak.

## Methods

Twenty three isolates of Kp from a hospital based Multidrug-Carbapenem resistant outbreak in 2010 at King Abdulaziz Medical city, Riyadh are the subject for study. MICs were determined by Microscan Walkaway system (Siemens) and confirmed using E-test (AB biodisk). DNAs were extracted using MagNApure kit (Roche’ Diagnostics). Sequence typing was performed according to Diancourt et al protocol for MLST. ESBL genes and genes for aac(6’)Ib, OqxB, qnrA, -B and -S were PCR amplified and sequenced as per published methods.

## Results

We found that four different clones of Kp are involved and ST-29 as the major clone (74%, 17/23) responsible for the outbreak. All isolates are positive for OqxB gene. Isolate #2 with ST-37 had maximum number of variations in their OqxB gene sequence resulting in change of amino acid Asparagine to Glycine at 148 and Arginine to Leucine at position 197 of the protein. There had been some variations in isolates 3, 6, 8, 19 and 22 but without any translational change. The isolates with ST-29 presented a normal OqxB gene. Those isolate with variations in their OqxB gene presented either SHV-11 or SHV-12 type of ESBL whereas the isolates with ST-29 exhibited SHV-1. Twenty out of 23 isolates were positive for qnrB gene. QnrA and –S genes were absent in all isolates. Two out of three isolates with ST-709 were associated with the absence of qnrB gene whereas isolate with ST-37 also lacked this gene. All isolates were positive for aac(6’)Ib except two which had ST-111.

## Conclusion

The data shows that different clone sequence types presented differences in the genetic make-up of their different resistance genes in Kp. The predominant clone type-29 in this outbreak presented a normal qnrB, OqxB, aac(6’)Ib in combination with SHV-1.

## Disclosure of interest

None declared

